# Caveats in reporting of national vaccine uptake

**DOI:** 10.7189/jogh.14.03006

**Published:** 2024-02-09

**Authors:** Tristan Millington, Kirsty Morrison, Karen Jeffrey, Christopher Sullivan, Amanj Kurdi, Adeniyi Francis Fagbamigbe, Ben Swallow, Ting Shi, Syed Ahmar Shah, Steven Kerr, Colin R Simpson, Lewis D Ritchie, Chris Robertson, Aziz Sheikh, Igor Rudan

**Affiliations:** 1 Usher Institute, The University of Edinburgh, Edinburgh, UK; 2 Public Health Scotland, Glasgow, UK; 3 School of Health, Wellington Faculty of Health, Victoria University of Wellington, Wellington, NZ; 4 Department of Mathematics and Statistics, University of Strathclyde, Glasgow, UK; 5 Strathclyde Institute of Pharmacy and Biomedical Sciences, University of Strathclyde, Glasgow, United Kingdom; 6 Department of Clinical Pharmacy, College of Pharmacy, Hawler Medical University, Erbil, Iraq; 7 College of Pharmacy, Al-Kitab University, Kirkuk, Iraq; 8 School of Pharmacy, Sefako Makgatho Health Sciences University, Pretoria, South Africa; 9 School of Medicine, Medical Sciences & Nutrition, Academic Primary Care, University of Aberdeen, UK; 10 School of Mathematics and Statistics, University of St Andrews, UK; 11 Algebra University College, Zagreb, Croatia

During the coronavirus disease 2019 (COVID-19) pandemic, policymakers and public health agencies urgently required contemporaneous data on vaccine uptake across whole populations and within their subsections. This was challenging because several doses of vaccine needed to be administered to different age groups at different times following their specific approval, and the number of doses may have also varied by specific subpopulations. There was a need for dynamic reporting among policymakers, health agencies, the media, and the general public. In this paper, we reflect on Scotland’s experience and report several caveats that must be considered when reporting vaccine uptake at a national level, especially when it is required for a specific age group, such as children or adolescents, or among older persons who are at particularly high risk.

In our recent paper on the BNT162b2 COVID-19 vaccination uptake, safety, effectiveness, and waning in children and young people aged 12–17 years in Scotland, we were asked by the reviewers to clarify our approach to measuring the uptake at different time points [1]. This is because the figures can be derived in almost real-time either from the national health registries or from electronic health records. Due to various circumstances which will be explained and discussed in this paper, those estimates may differ at the same point in time, which has to be considered when the rates of uptake are later used in rigorous research.

## APPROACHES TO MEASUREMENT: VACCINATION REGISTRIES AND ELECTRONIC HEALTH RECORDS

Conceptually, vaccine uptake should be straightforward to measure. It is simply the number of people vaccinated (the numerator) over the total eligible population (the denominator). However, this can become challenging in practice for several reasons which are rarely discussed in the literature [2]. First, the number of vaccinated people should be traceable in any given population or a country through, for example, a national health registry, electronic health records, or a combination of the two, as is the case in Scotland. The registry should match the serial number of each vaccine dose with a national identifier or health insurance number, as applicable, of its recipient. Therefore, if the registry is established robustly and information is centralised or accessible from a federated system, then a precise number of vaccinated individuals can, in theory, be determined for any given period. However, these registries often differ across the world – some countries do not have such registries, while others store their data in separate, siloed databases.

## POPULATION DENOMINATOR

Accurate population denominator data are needed to determine the proportion of individuals vaccinated. However, problems arise in the real world because the denominator is not clearly defined or static. For example, it may be unclear because it depends on the latest population census, which is typically outdated and misrepresents the actual number of a country’s current residents. Additionally, the number of people with health insurance, where that provision is required, may differ in any country at a given date from the number of people living in that country, even when the census is very recent and deemed to be sufficiently accurate. This may be due to both considerable emigration from the country with health insurance status preserved and/or larger immigration from other countries with health insurance granted. Likewise, the denominator is dynamic rather than static: for any specific age group, some people enter and some leave that specific age group throughout the calendar year. Vaccination campaigns take time to be rolled out, so the composition of any age group in the population will be different at the end of the campaign in comparison to the beginning.

An additional problem can arise if there are reasons for a subset of the population within a specific age group to be vaccinated even before the official vaccination programme begins, where inevitable idiosyncrasies pose a further challenge. For instance, in the case of the COVID-19 vaccination rollout in Scotland [1], some children and young people received their first vaccine dose before the official programme started – either because they had a special condition that placed them at higher risk, or to protect a high-risk member of the household.

## THE SCOTTISH EXAMPLE: IDENTIFYING CHILDREN AND YOUNG PEOPLE (AGED 12–17) WHO RECEIVED COVID-19 VACCINATION(S)

From our experience with COVID-19 vaccine uptake in children and young people (aged 12–17 years) in Scotland, assessing who had been vaccinated (i.e. the numerator) was reasonably straightforward. The information was provided by the Turas Vaccination Management Tool [3], which vaccinators use to record information and whose data are available in a centralised system. Eligibility was based on the advice provided by the UK’s Joint Committee on Vaccination and Immunisation. It was primarily based on age [4], with younger age groups becoming eligible for vaccination later in the pandemic. The exact dates at which specific vaccines were recommended to age groups and how many doses are available from the COVID-19 vaccination programme timeline for Scotland [5]. The challenge was determining the size of the eligible population (our denominator).

### Differences between the demographic population estimates and the number of health service users

The main issue with determining the eligible population was deciding who was resident in Scotland [1]. The Early Pandemic Evaluation and Enhanced Surveillance of COVID-19 (EAVE II) platform has been set up to allow surveillance of respiratory pathogens at the national level based on digital information, so it was ideally placed during the pandemic to provide information on COVID-19 in almost real-time [6]. However, even under such favourable circumstances, the platform still had strict guidelines on what data could be stored and used for research and surveillance, which impacted uptake measurement, particularly with restrictions on recording age and ethnicity. The EAVEII dataset contained 5.7 million people (comprising all patients registered with every general practice in Scotland), despite the population of Scotland being 5.4 million [1]. It is therefore possible that some patients will have moved and registered with a new general practice without their previous registration being cancelled, while others may have left Scotland, again without cancelling their previous general practice registration. Both of these have inflationary effects on patient numbers and have been referred to as ‘ghost patients.’ This is independent of their vaccination status.

The census in Scotland before the EAVE II cohort was carried out in 2011; population projections since then have usually been based on the mid-year estimates of the Office for National Statistics (ONS) [7] rather than a census. The latest census was then carried out in 2021. Moreover, National Records of Scotland (NRS) mid-year population estimates are always reported for an earlier year [8]; they are initially based on the ONS estimates [7], but are then extrapolated based on births, deaths, and immigration data [8]. Therefore, the total populations for 12–17-year-olds in Scotland in EAVE II, NRS, and ONS are all likely to be slightly different and somewhat imprecise, with the EAVE II figure likely being the largest of the three and a slight overestimate.

It is therefore not impossible that some individuals were no longer residents of Scotland, but were still considered a part of its population and a denominator for the uptake assessment. Some could have also been vaccinated outside of Scotland. Importantly, EAVE II participants were patients registered in all general practices in Scotland at any given point [6]. This would certainly contribute to an inflated figure, as some patients may have moved to other practices within or without Scotland in the UK or moved abroad, without any change in their original registration status.

To attempt to address this issue, we used weighting. We gave more weight to individuals who had interactions with the health care service in the recent past, i.e. years immediately preceding the vaccination programme. This correction was easier to perform among the vaccinated, who had an interaction with the health care service at the point of their vaccination, than among those unvaccinated. This method consequently had some limitations, because it is likely to pick up those who have ill health and are more likely to be vaccinated, thus potentially introducing bias towards those with underlying health conditions. A sensitivity test could be performed for that possible caveat through a correction for the persons with previous recent interactions, but excluding those with vaccination appointments.

The cumulative size effects of the discrepancies between different sources observed in Scottish data have never reached double-digits in absolute, population-level coverage (which ranges from 0% to 100%) at any point in time during the pandemic, meaning that we could have encountered discrepancies of less than 10% (e.g. 47% vs 53%). However, it is possible to imagine that the differences could be greater at some points in time in different countries, or under different circumstances – for example, when there is a longer time lag of reporting to national health registries while the data from electronic health records are available in near real-time, or when a cohort-based approaches give different estimates than those based on an estimated “average” population during a time period.

### Misclassification due to unavailable date of birth

In the EAVE II project, we did not have access to the exact date of birth of any person – an issue that is likely to become increasingly common with the introduction of the General Data Protection Regulation-related legislation in Europe [8]. Instead, we had data on individuals’ age on 1 March 2020, which meant that the recorded age could be up to a year wrong if the vaccinated person was born on 2 March. This is an issue when deciding if an individual fits into the 12–15 or 16–17 years age groups on a given date, or if they may turn 18 years. The extent of this error also depends on how the ages were derived on 1 March. If it was a ‘round down’ to the nearest integer, then the errors could be larger. If a simple rounding was done instead, then the error would have been up to six months, and not one year.

Our initial data linkage in EAVE II was facilitated by the Community Health Index (CHI) number, which did contain the individual's date of birth; however, its use was restricted to the purpose of linkage only. One way in which we could control for possible errors was the information on the ‘age at vaccination,’ which was available for vaccinated individuals. However, this still did not help us for those who did not get vaccinated. Therefore, we advocate that an estimate of this error could be reported in the papers on vaccine uptake by showing the proportion of those for whom the actual age was misclassified.

### Vaccination before being eligible

As mentioned earlier, we also encountered the issue of individuals vaccinated before being eligible based on the scheduled rollout with a vaccine that was licensed for their specific age group, which would affect the numerator again. In Scotland, there were children and young people either with certain comorbidities or who lived in households with certain other vulnerable people who received vaccine doses before becoming eligible for the official programme. Deciding how to include these children and young people in the uptake reporting and whether to break them out into a separate category or merge them with those who were vaccinated late with a vaccine of different composition was a moot point.

### Discrepancies between national-level vaccine uptake reports from different sources

Based on our experience in Scotland, the main challenge with reporting the uptake of COVID-19 vaccines was determining the size of the unvaccinated population [9] and therefore the denominator in the uptake formula. This was due to a dynamic situation concerning the population’s size, coupled with not having the date of birth of each person available, not knowing exactly who resided in Scotland at a given time, and deciding how to handle those vaccinated early. The lack of ethnicity data in Scotland was also an issue, although the 2022 census results should help us for future work.

For all the above-mentioned reasons, both the numerator and denominator data used in the EAVE II study to compute the vaccine uptake could have differed from those used by Public Health Scotland (PHS) at any given date. PHS reported only the number of vaccinated persons, thus avoiding the problem of comparability. This also meant that they could avoid using a specified cohort, so they could have included vaccinated persons who were not part of the EAVE II cohort – e.g. those temporarily in Scotland. Still, having a specified cohort defined – for example, those born in a specific year – makes it easier to have a denominator than operating in a time window of an ongoing vaccination programme and deciding what the denominator is for a specified age group, as EAVE II needed to do in real-time, leading to potential discrepancies in comparison to other national estimates.

## DISCUSSION

Although the rate of uptake of a specific vaccine at the national level seems simple to compute and report in theory, several caveats should be considered when measuring it at the population level, especially if this needs to be done for a specific age group:

Data accuracy and reliability: The accuracy and reliability of the data on vaccination and on the eligible population are essential for the correct computation. Data collection methods and reporting systems need to be accurate and robust. Inaccurate or incomplete data on either the vaccination or the eligible population can lead to misleading conclusions about vaccine uptake rates.

Variations in data collection methods: Different regions or sub-populations may employ different methods for collecting vaccination data, leading to inconsistencies in measuring vaccine uptake. For example, some areas may rely on self-reporting, while others may use proper administrative records or digitalised records. These variations can affect the comparability of data between different regions, lead to changes in reported rates over time, and should all be considered.

Vaccination coverage definitions: The definition of vaccine uptake can vary depending on the specific age group and the target population being considered, as in the case of 12–17 years in Scotland discussed here. Some measurements may focus on the receipt of a single dose, while others may require completion of the full vaccination schedule and boosters in some cases. It is crucial to understand the specific definition being used to interpret and compare the results accurately.

Age misclassification: As shown here, determining the exact age of individuals in the population can sometimes be challenging, especially when it is done on a large scale. Age misclassification can occur due to lack of access to date of birth, limited data on age at vaccination, errors in data recording, or discrepancies between self-reported age and official records. This caveat is an important, specific component of the larger issue of data accuracy and reliability.

Discrepancy between definitions: The denominator can be defined as the average number of persons within an age group during the time window of a vaccination programme. However, a birth cohort approach can also be used. The numerator may also be monitored in real-time and updated immediately, or it could be increased periodically. This can affect the accuracy of vaccine uptake calculations in specific age groups due to differences in both the numerator and the denominator.

Vaccine availability and accessibility: Vaccine uptake may be influenced by factors such as vaccine availability and accessibility, especially when the report is required at the national level. There will be large variations in vaccine supply, distribution, and accessibility across different regions or populations. This was a particular issue during COVID-19, as there was a need for real-time uptake figures, which could further highlight these inequalities. If they are not considered and corrected for, those differences can have a considerable impact on the measurement of vaccine uptake at the population level, particularly in marginalised and/or remote communities.

Population dynamics and demographic factors: One special case of unintentional and partial age misclassification is related to the changes in the vaccinated cohort’s composition during the vaccination programme. The composition of the population within a specific age group can change over time due to factors such as migration, birth rates, or ageing populations and deaths. These demographic shifts can affect the interpretation of vaccine uptake rates, as the denominator – which is the total population in the age group – may vary from the start to the end of the vaccine coverage programme. Even in a situation where we could estimate both the numerator and the denominator correctly, ethnicity data might still be unavailable or unreliable. This is an issue that came up for the Scottish data. Ensuring equitable access to vaccination is important to reduce health inequalities, and there has also been considerable evidence that the risk of COVID-19-related hospitalisation or death varies by ethnic group [10]. Being able to look at the uptake across Scotland’s different ethnic groups would have been very useful, but unfortunately, we could not reliably do this in our work.

Bias and confounding factors: When measuring vaccine uptake, factors such as socio-economic status, education level, cultural beliefs, and vaccine hesitancy can impact vaccine uptake rates. These factors need to be carefully considered, because their neglect may lead to misinterpretation of the data. As an example, if the vaccine uptake is based on self-reporting using a large sample from the total national population, it should be ensured that the sample matches the general population in all the factors that may affect vaccine uptake.

Data privacy and ethics: When collecting and analysing data on vaccine uptake, it is crucial to ensure compliance with privacy regulations and ethical considerations. Confidentiality and data protection must be maintained to safeguard individual privacy rights.

Any other real-world issues: Any departures from the expected and recommended practices which could also affect the reported uptake need to be carefully documented and transparently presented. An example in Scotland was the case of vaccinating some of the 12–17-year-olds before the programme for children and young people officially started, to protect the children and young persons who were especially vulnerable or their household members.

To address and mitigate all these caveats, researchers and public health authorities should employ standardised data collection methods, ensure data accuracy and completeness, consider demographic factors and potential biases, and employ rigorous statistical analysis techniques. Adherence to this ‘checklist’ could improve the reliability of vaccine uptake measurements at the population level. There is a need for an iterative process and triangulation, offering opportunities for partial validation and replication, which are crucial under these circumstances. We underscore the imperative for cohesive efforts and harmonisation, particularly in high-crisis scenarios, and summarise the caveats and present a unified checklist for reporting national-level vaccine uptake at specific dates and in specific age-groups in research reports (**Box 1**).

Box 1 **A summary of caveats and a unified checklist for reporting national-level vaccine uptake at specific dates and in specific age groups in research reports.**

1. What is the source of information on vaccine uptake: National health registry/electronic health records/paper records stored in a separate database/other/multiple sources?
2. If multiple sources are consulted and used to assess vaccine uptake, what are the levels of discrepancy observed between different sources at specific dates? Can the discrepancies be quantified and studied over time? What are the most likely causes of those discrepancies?
3. What is the exact source of data on the population denominator? How recent is the estimate of the total population? Are there multiple sources that can be used for the denominator (e.g. national population statistics/health insurance users/persons registered in primary health care/other/multiple sources)?
4. If multiple sources are consulted and used to assess population denominator, what are the levels of discrepancy observed between different sources? Can the discrepancies be quantified and studied? What are the most likely causes of those discrepancies?
5. What is the justification for choosing the most reliable source of data for the numerator (i.e. the number of vaccinated individuals at any given point in time in the country) and for the denominator (e.g. the number of individuals living in the country at that same point in time) when multiple sources are available? What is the likely direction of bias for the numerator and the denominator?
6. Can an assessment be provided for the possible effects of misclassification in both numerator and denominator? Can the most likely causes of such misclassification be identified and reported, and the size and direction of the effect assessed?
7. Can an assessment be provided of the possible effects of different eligibility for vaccination for specific sub-populations over time, different timing of vaccination programmes for different age groups, differences in the dose and composition of vaccines applied to specific sub-populations, differences in vaccine types and manufacturers of vaccines, and any other technical reasons that could have affected the uptake of vaccines at the national level?
8. Are there any reasons to believe that the source of data on those who are vaccinated at some point in time may be inaccurate, incomplete, or unreliable? What could be the reasons for this? Was it possible to carry out an iterative process and triangulation, offering opportunities for partial validation and replication?
9. Has more weight been given to individuals who had interactions with the healthcare service in the years immediately preceding the vaccination programme? Could this potentially introduce bias towards those with underlying health conditions? Could a sensitivity test for this possible caveat be performed?
10. Are there reliable national-level data on, for example, ethnicity, gender, socio-economic differences, education level, occupation, and others, that could be used to study whether the uptake is equitable and the access to vaccination universal, or are there clear inequalities in vaccine uptake? If there are inequalities, what are the main drivers?
